# The impact of the COVID-19 pandemic on patients’ experiences obtaining a tuberculosis diagnosis in Peru: a mixed-methods study

**DOI:** 10.1186/s12879-022-07832-2

**Published:** 2022-11-09

**Authors:** Ana Karina Millones, Leonid Lecca, Diana Acosta, Hortencia Campos, Erika Del Águila-Rojas, Sheyla Farroñay, Giannina Morales, Judith Ramirez-Sandoval, Isabel Torres, Judith Jimenez, Courtney M. Yuen

**Affiliations:** 1 Socios En Salud Sucursal Peru, Lima, Peru; 2 Department of Global Health and Social Medicine, Harvard Medical School, Boston, MA USA; 3 Division of Global Health Equity, Brigham and Women’s Hospital, Boston, MA USA

**Keywords:** Tuberculosis, SARS-CoV-2, Delayed diagnosis, Costs and cost analysis

## Abstract

**Background:**

The COVID-19 pandemic disrupted TB services worldwide, leading to diagnostic delays. There have been few published reports describing how the pandemic affected people’s pathway to diagnosis from their own perspectives. We sought to evaluate the impact on the pandemic on people’s experiences obtaining a TB diagnosis.

**Methods:**

We performed a mixed-methods study, enrolling newly diagnosed TB patients from 12 health centers in Lima, Peru. We used structured surveys to quantify diagnostic delay, defined as the time between symptom onset and diagnosis, and in-depth interviews to understand the ways in which the pandemic affected the pathway to care. We compared diagnostic delay between patients enrolled during the first year of the pandemic to those diagnosed after using a Wilcoxon rank-sum test. We used an inductive content analysis approach to analyze interview content related to the pandemic.

**Results:**

We enrolled 51 patients during November 2020–April 2021 (during the first year of the pandemic) and 49 patients during October 2021–February 2022. Median diagnostic delay was longer for patients diagnosed during the first year of the pandemic (median 15 [IQR 5–26] weeks compared to 6 [IQR 3–18] weeks, p = 0.027). Qualitative analysis of 26 interviews revealed that the pandemic affected participants’ care-seeking behavior and their ability to access to TB diagnostic services, particularly for those diagnosed in the first year of the pandemic. Many participants initially had their symptoms attributed to COVID-19, resulting in delayed TB evaluation and additional costs for COVID-19 treatment.

**Conclusions:**

The COVID-19 pandemic impacted multiple steps in the pathway to care for TB patients in Lima, causing delays in TB diagnosis. These findings demonstrate how the shifting of health care resources to prioritize COVID-19 can lead to collateral damage for people with TB and other conditions.

**Supplementary Information:**

The online version contains supplementary material available at 10.1186/s12879-022-07832-2.

## Introduction

The global COVID-19 pandemic overwhelmed and disrupted health services worldwide, negatively impacting the delivery of TB services in many countries. Globally, there were 18% fewer TB cases diagnosed in 2020 compared to 2019 [1], and reports from diverse countries have measured significantly longer delays in TB diagnosis during the pandemic [2–5]. Modeling studies suggest that the pandemic has caused substantial increases in TB-associated mortality by delaying TB diagnoses or causing diagnoses to be missed completely [6, 7].

Despite the abundance of quantitative evidence of the pandemic’s impact on TB diagnoses, there have been few published reports describing the ways in which the pandemic affected people’s pathway to diagnosis from their own perspectives. Qualitative studies exploring patients’ experience with TB during the pandemic have reported patients’ concerns around the financial and social costs of lockdowns, their ability to continue accessing TB medications, and their fear of infection [8–11]. However, these studies have not identified ways in which the pandemic had impacted patients’ experience obtaining a TB diagnosis.

Understanding how the COVID-19 pandemic affected people’s pathways to TB diagnosis is important for improving the resiliency of health systems so that future public health emergencies do not have such a damaging impact on TB detection. We therefore sought to understand the perspective of people with TB on how the pandemic affected their ability to be diagnosed with TB. We performed a mixed methods study in Peru, a country with a high TB burden, which experienced serious morbidity and mortality as a result of the COVID-19 pandemic.

## Methods

### Study design

Prior to the pandemic, we designed a sequential explanatory mixed-methods study to understand barriers to prompt TB diagnosis in Lima, Peru. However, after enrolling half the cohort of TB patients during the first year of the pandemic, we realized that the pandemic’s effect on the health system was causing delays in diagnosis. Therefore, we delayed enrollment of the second half of the cohort until a year after the study start date in hopes that health services would have normalized, allowing us to quantify the effect of the acute stage of the pandemic. We performed quantitative analysis of surveys to assess differences in TB diagnostic experiences between the two periods and qualitative analysis of interviews to explain these differences. Although the original study design was sequential, we used a convergent approach to integrating quantitative and qualitative information on the impact of the pandemic since this was not an original study objective and the quantitative results did not inform qualitative data collection on this topic.

### Study setting

Our study took place in Carabayllo, a district on the periphery of Peru’s capital Lima. Peru has an estimated TB incidence of 116 per 100,000 population and the second-highest TB burden in the Americas. Peru’s health system was greatly impacted by the COVID-19 pandemic [12], with health care visits for other conditions decreasing by 64% [13] and TB diagnoses decreasing by 25% in 2020 [1]. The first case of COVID-19 was detected in March of 2020, with increasing cases by April. The first wave of pandemic peaked in August 2020 and the second wave in March-April 2021 [14]. Vaccines became available in February 2021, and half the population had received at least one dose by September [15]. As of May 2022, Peru had experienced the highest reported cumulative population mortality rate from COVID-19 in the world, with deaths concentrated during the first and second waves, prior to widespread vaccine availability [16].

### Quantitative data collection and analysis

We enrolled a convenience sample of 100 adults who had recently enrolled for TB treatment at the 12 Ministry of Health primary care facilities in Carabayllo, purposively distributing enrollments across the different facilities. Patients were eligible for enrollment if they were ≥18 years old and had started TB treatment within the last month. We enrolled half the sample during November 2020–April 2021 (period 1) and half during October 2021–February 2022 (period 2). Period 1 was during the first year of the pandemic in Peru, encompassing the first and second waves of COVID-19. Period 2 was after widespread vaccine availability.

Structured surveys asked participants when they first felt sick with their current episode of TB and about each visit to the health system until the point where they were diagnosed with TB. For each visit, participants were asked the date of the visit and the amount of money that they spent on transport, medical procedures, and drugs. For each participant, we calculated (1) total diagnostic delay, defined as the number of weeks between symptom onset and diagnosis, (2) delay before contact with the health system, defined as the weeks between symptom onset and the first visit to a health facility, and (3) delay after contact with the health system, defined as the weeks between the first visit to a health facility and diagnosis. We also calculated total out-of-pocket expenditures, using an exchange rate of 3.7 Peruvian soles to 1 USD. We used a Wilcoxon rank-sum test to assess differences in delay and expenditures between periods, sexes, and age groups (18-34 years old versus ≥35 years old). Analysis was performed in SAS v9.4 (SAS Institute, Cary, NC).

### Qualitative data collection and analysis

We recruited participants for in-depth interviews based on the delays that they reported during the surveys, balancing the sample in terms of long versus short delays before contact with the health system, and long versus short delays after first contact with the health system. The study team member who conducted the survey recruited participants for one-time interview lasting approximately 1 h. One or two study team members conducted each interview in Spanish in the health center or the patient’s home during April–May 2021 and November 2021–January 2022. All interviewers (DA, EA, HC, SF, GM, JR, IT) were female Peruvian nurse technicians who were trained in interviewing and had no prior relationship with the participants. We interviewed 16 participants from period 1 (participants #1-16) and 10 from period 2 (participants #17-26). Twelve individuals declined interviews upon recruitment; none withdrew during the interview.

The interview guide specified probes based on the information reported during the survey, asking participants about initial symptoms, what prompted them to seek care in each instance, and what happened during each health facility visit. After recounting this pathway to diagnosis in detail, participants were asked either what factors they think led to prompt care-seeking or diagnosis or what factors they think delayed their care-seeking or diagnosis. They were also asked what interventions they believed could encourage prompt diagnosis for others in the future. We did not specifically ask about COVID-19. We planned to interview 30 patients but stopped after 26 because no new themes were emerging relating to factors that facilitated or delayed diagnosis, which we considered a sign of data saturation. Interviews were audio recorded, transcribed, and checked by the interviewer for fidelity with the help of field notes; transcripts were not returned to participants.

We used an inductive content analysis approach [17] to understand the impact of the COVID-19 pandemic on TB diagnosis. Two authors (AKM, CMY) with experience in qualitative research open-coded content related to COVID-19 or the pandemic in five transcripts (in Spanish), resolved discrepancies by consensus, and applied the resulting codebook to the remaining transcripts. Coding was done manually in Microsoft Word. We analyze the coded data, grouping themes into higher-level categories using an iterative approach. Findings were not shared directly with participants but will be disseminated via public community-oriented presentations. Strategies to ensure analytic rigor included having two coders independently performing the initial open-coding to develop the codebook, discussing interpretations with co-authors who performed the interviews (DA, SF, IT) as well as co-authors who were not part of the interview process (AKM, LL, CMY), and using rich verbatim data from patient interviews to illustrate findings.

## Results

### Comparing diagnostic delay between two periods

We enrolled 51 participants during period 1 (November 2020–April 2021) and 49 participants during period 2 (October 2021–February 2022). Participants were enrolled from all 12 health centers in Carabayllo district, with a median of 8 patients (range 4–14) per health center. Participants characteristics are shown in Table 1.

**Table 1:** Characteristics of patients with tuberculosis recruited for surveys (N=100) and in-depth interviews (N=26) in Lima, Peru

	Surveys (N=100)		Interviews (N=26)	
	n	(%)	N	(%)
*Sex*				
Female	41	(41)	14	(54)
Male	59	(59)	12	(46)
*Age*				
18–34	54	(54)	15	(58)
35–59	25	(25)	7	(27)
60+	21	(21)	4	(15)
*TB type*				
Pulmonary	80	(80)	18	(69)
Extrapulmonary	20	(20)	8	(31)

Participants enrolled during period 1 reported a median delay between symptom onset and diagnosis of 15 weeks (IQR 5–26) (Table 2). Those enrolled during period 2 reported a median delay of only 6 weeks (IQR 3–14), and the difference was statistically significant (p=0.027). Participants diagnosed during period 1 also reported spending significantly more in obtaining their diagnosis (p=0.009). Neither sex nor age was significantly associated with total delay, delay before contact with the health system, or delay after contact with the health system. Delay before contact with the health system was not associated with delay after contact with the health system.

**Table 2:** Diagnostic delays and out-of-pocket expenditure reported by survey participants (N=100)

	Period 1* median (IQR)	Period 2* median (IQR)	Wilcoxon rank sum p-value for comparison of period 1 vs 2
Total weeks of diagnostic delay	15 (5–26)	6 (3–14)	0.027
Weeks of delay before contact with health system	4 (0–10)	4 (0–8)	0.497
Weeks of delay after contact with health system	4 (0–10)	2 (0–6)	0.165
Number of visits to health system	2 (2–4)	3 (2–5)	0.029
Amount spent on transport, visits, and medications (USD)	80 (13–222)	22 (4–61)	0.009

^*^Period 1 (November 2020–April 2021) was during the first year of the COVID-19 pandemic in Peru; Period 2 (October 2021–February 2022) was after most of the country had been vaccinated

### Effect of the pandemic on TB diagnosis

**Fig. 1:**
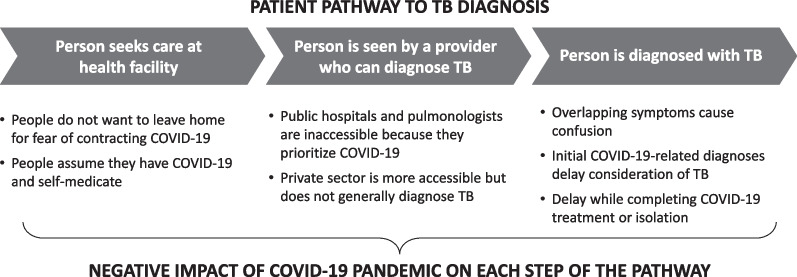
Impact of COVID-19 pandemic on the pathway to TB diagnosis

Interview participant characteristics are shown in Table 1. Qualitative analysis revealed that the pandemic affected participants’ care-seeking behavior and their ability to access to TB diagnostic services, particularly for participants in period 1 (Fig. 1, Additional file 1: Tables S1, S2). Once they accessed health services, many participants in both periods initially had their symptoms attributed to COVID-19, resulting in delayed TB evaluation and additional costs for COVID-19 treatment (Additional file 1: Table S3).

#### The pandemic affected care-seeking behavior

Participants described ways in which the pandemic could have delayed their seeking care for their symptoms. Some mentioned a reluctance to leave the home for fear of contracting COVID-19. Others reported initially self-medicating what they believed were COVID-19 symptoms.

“I am afraid when I leave my house, as I suffer from asthma. Thank God we still have not caught [COVID-19] in my house. But my neighbors, it has already swept through for most of them, and there are some who have died. In my aunts’ house too, it has swept through my family. We just stay in my house, thank God. That’s why we don’t go out – I have my grandparents there who are old, and if we go out we can infect them.”

(Participant #4, female, 35-59 years old)

“I was feeling bad, but my brother was self-medicating me. He was giving me pills like ‘Nastizol’ and ‘Nastiflu’ to control COVID.” (Participant #24, male, 35-59 years old)

#### The pandemic reduced access to TB diagnostic services

The pandemic affected the health system, reducing participants’ ability to access TB services. Participants described how they were unable to receive care because health facilities, particularly public hospitals, were focused on managing COVID-19 patients and consequently cut back other services. Participants also described being unable to get an appointment with a pulmonologist because the pulmonologists were working exclusively with COVID-19 patients. One participant felt that the doctor at his health center did not want to see him for fear that his symptoms might indicate COVID-19. While these experiences were described by participants in both periods, they were particularly pronounced for participants in period 1.

“From the [public health system] I did not receive good care. I was waiting there almost half a day, and no one gave me any information. They did not want to help me because everything was totally COVID.” (Participant #11, male, 18-34 years old)

“In the hospital I was delayed so much because I could not get an appointment. One day they sent me for an appointment, but then there wasn’t one. So I had to wait and re-book the appointment. They gave me a pulmonology appointment for [almost 2 months later] because there were no appointments, there were no doctors. Because of the pandemic, everything was restricted” (Participant #21, female, 35-59 years old)

Some participants described seeking care in the private sector, in some instances after failing to receive service in the public sector. However, while the private sector may have been more accessible, the private sector in Peru does not generally perform complete TB evaluations with sputum testing. Participants who had chest radiographs read as abnormal in private clinics reported being diagnosed with COVID-19 or being referred to the public sector for TB evaluation.

“All the [public] health posts, the hospitals, they were all practically closed because they only attended to people with COVID; they were closed. I went to [a public-private partnership hospital], but it was closed – they would not see patients. And I went to one of those [private] medical centers around that area, and there they gave me a chest x-ray.” (Participant #2, male, 18-34 years old)

#### Participants received COVID-19-related diagnoses, causing additional delays and costs

Participants noted that the overlap between TB and COVID-19 symptoms was confusing to both doctors and patients. Several reported having initially been diagnosed with COVID-19 based on symptoms and/or abnormal chest radiography and given ivermectin or other treatments. This happened even for those who received a negative SARS-CoV-2 test result. Some reported doubting that they had COVID-19 but nonetheless accepting the diagnosis. A couple participants reported that doctors attributed their symptoms to side effects of the COVID-19 vaccine. After a COVID-19 diagnosis, participants reported further delays while they completed isolation or treatments prescribed for COVID-19. They also reported paying for these treatments out of pocket.

“The only symptom I had until then was a cough that would not go away, it would not go away with anything. And that is why I went - I said, ‘Wow! I do not think this is COVID because I do not have a headache, body ache, or anything else.’ Well, I went, and the doctor prescribed me these medications for COVID – ivermectin and all these things. And well, I took them, but I went on in the same way – they didn’t do anything for me. On the contrary, I think I got worse.” (Participant #2, male, 18-34 years old)

“I took the treatment, but this treatment was extending to a week – that is, from three days to a week – and on top of that more medications, and on top of that, every time I had to pay.” (Participant #6, male, 18-34 years old)

#### Applying lessons from the COVID-19 response to TB

When asked for suggestions of how to reduce TB diagnostic delays, multiple patients mentioned that there should be public awareness campaigns to increase knowledge of TB symptoms, similar to what had been done for COVID-19 (Additional file 1: Table S4).

“They should make campaigns that are shown on television, just like there are for COVID. They should also do this for TB, which is a disease – they should explain what the symptoms are and everything, how it is treated.” (Participant #8, female, 18-34 years old)

## Discussion

The COVID-19 pandemic impacted multiple steps in the pathway to care for TB patients in Lima, causing longer delays in TB diagnosis during the first year of the COVID-19 pandemic compared to after. People diagnosed during the first year of the pandemic also spent more money on accessing care, with participants describing out-of-pocket payments for COVID-19 treatments and attention in the private sector. Within the health system, the focus on the pandemic led to potentially incorrect COVID-19 diagnoses and delayed consideration of TB, as well as reduced access to hospitals and pulmonologists. As the health system recovered in the second year of the pandemic delays in TB diagnosis returned to pre-pandemic levels [18], although confusion of TB and COVID-19 signs and symptoms still complicated the diagnostic process.

Our qualitative findings show how the pandemic’s impact on Peru’s health system forced people affected by TB to suffer through prolonged illness, repeated attempts to access diagnostic services, and misdiagnoses. While we are not aware of other qualitative studies focused on the pandemic’s impact on patients’ experiences during the TB diagnostic process, patients who were already receiving treatment for TB have reported difficulty accessing health services for treatment monitoring [10]. In addition, a survey of health care workers from 64 countries found that most felt that people with TB and HIV faced greater challenges accessing health services during the pandemic, with reduced mobility and health facility closures frequently stated as reasons [19]. While the pandemic’s effects on TB-associated mortality have not yet become clear [20], increased deaths from cardiovascular disease [21, 22], neonatal deaths [23], and deaths attributable to delays in accessing medical care during the pandemic [24] have been reported in various countries. Thus, it is an important lesson for future public health emergencies that changes to health system priorities and procedures should consider who may be harmed as well as who will benefit.

Our finding that the initial phase of the COVID-19 pandemic was associated with longer delays in TB diagnosis is consistent with reports from other countries that showed that patients experienced longer overall delays during the pandemic compared to before [2–5]. In most studies that distinguished between delay before and after contact with the health system, the former was far longer than the latter; in these studies, the average delay after entering the health system was generally under a week, even during the pandemic, and overall delay was driven by the delay in accessing health services [2, 3, 5]. However, in our study, many patients experienced substantial delays in receiving a TB diagnosis after accessing health services—an average of 4 weeks during the first year of the pandemic and 2 weeks in the period after. Interestingly, a study from Burkina Faso found that the average time to TB diagnosis once people entered the health system decreased substantially during the pandemic, with possible explanations including increased access to molecular diagnostics and increased TB screening as a means of ruling out a COVID-19 diagnosis [25]. Thus, it is possible for a public health emergency such as the COVID-19 pandemic to have beneficial effects for people with other health conditions if the emergency response ultimately strengthens the health system.

The difficulty of differentiating TB from COVID-19 symptoms and the instances of potentially incorrect COVID-19 diagnoses reported by participants in our study suggests the importance of integrated evaluation in places where both conditions are common. Models for integrated screening have been reported from multiple countries [26–28], and the Peruvian Ministry of Health has recently established an integrated evaluation algorithm for people with respiratory symptoms. Implementing integrated programs requires not only training providers in these algorithms, but also investment in robust testing capacity for both conditions. In particular, access to rapid SARS-CoV-2 testing is necessary so that a negative test can quickly help doctors focus on alternative diagnoses.

The major limitation of our study is that it was not originally designed to assess the impact of the COVID-19 pandemic on TB diagnosis, so this question was not specifically probed during interviews. However, the fact that so many patients described ways in which the pandemic impacted their pathway to diagnosis without being asked underscores the importance of our findings. Another limitation is that quantitative analyses of diagnostic delay are dependent on patient recall of when symptoms started, and recall can be imperfect, particularly when symptoms started a long time before diagnosis. However, the in-depth interviews conducted corroborated the details of the pathway to care for a large subset of patients, suggesting that recall of this major life experience is accurate immediately following diagnosis. Finally, we did not collect data on many patient characteristics (e.g. income, specific symptoms) in the quantitative survey, limiting our ability to assess differences between patient groups.

In conclusion, our findings serve as a warning about unintended negative effects of health system responses to the COVID-19 pandemic on people affected by TB. In addition, they suggest several ways in which services can be improved. The information dissemination methods used to rapidly create high public awareness about COVID-19 symptoms could be used to improve TB awareness and promote care-seeking behavior. In addition, training doctors in both the public and private sectors to use clear diagnostic algorithms for people with respiratory systems can help avoid delay in considering a TB diagnosis. As countries exit the acute phase of the COVID-19 pandemic, it is important to rebuild health systems to restore and improve services for TB and other conditions while maintaining capacity to manage COVID-19.

## Supplementary Information

**Additional file 1: Table S1.** Example quotations showing how the pandemic affected care-seeking behavior. **Table S2.** Example quotations showing how the pandemic reduced access to diagnostic services. **Table S3.** Example quotations showing how patients received COVID-19-related diagnoses, leading to delays and costs. **Table S4.** Example quotations showing how patients felt that tactics for raising COVID-19 awareness should be applied to TB.

## Data Availability

All data supporting the current study are available in the supplemental files or the Harvard Dataverse repository (https://doi.org/10.7910/DVN/ZDMH23).
