# Supplementary material for: The impact of the COVID-19 pandemic on patients’ experiences obtaining a tuberculosis diagnosis in Peru: a mixed-methods study
Source: BMC Infect Dis. 2022 Nov 9;22:829. doi: 10.1186/s12879-022-07832-2 (PMC9645304; doi:10.1186/s12879-022-07832-2)
Supplement: Supplementary file 1 — Additional file 1: Table S1. Example quotations showing how the pandemic affected care-seeking behavior. Table S2. Example quotations showing how the pandemic reduced access to diagnostic services. Table S3. Example quotations showing how patients received COVID-19-related diagnoses, leading to delays and costs. Table S4. Example quotations showing how patients felt that tactics for raising COVID-19 awareness should be applied to TB. [file 12879_2022_7832_MOESM1_ESM.pdf]

Table S1: Example quotations showing how the pandemic affected care-seeking behavior

Theme	Example quotation	Patient
Patients self-medicated for COVID-19 before seeking care at a health facility	They said I had to go to a hospital so that they could put me on medications, but I didn't go – I went to a pharmacy, and the doctor put me on medications.	Patient #15, female, 18-34 years old, pulmonary TB
	I was feeling bad, but my brother was self-medicating me. He was giving me pills like "Nastizol" and "Nastiflu" to control COVID.	Patient #24, male, 35-59 years old, pulmonary TB
Patients fear contracting COVID-19, which might reduce their willingness to seek medical care	I am afraid when I leave my house, as I suffer from asthma. Thank God we still have not caught [COVID-19] in my house. But my neighbors, it has already swept through for most of them, and there are some who have died. It my aunts' house too, it has swept through my family. We just stay in my house, thank God. That's why we don't go out – I have my grandparents there who are old, and if we go out we can infect them.	Patient #4, female, 35-59 years old, pulmonary TB
	[When asked why people might not attend a free community-based TB screening site] Some people are working. Some people have relatives with COVID, and because they are there supporting and helping them, I think they do not go. So because of lack of time, and also for fear of contracting COVID.	Patient #8, female, 18-34 years old, pulmonary TB

Table S2: Example quotations showing how the pandemic reduced access to diagnostic services

Theme	Example quotation	Patient
Health facilities were only attending to people with COVID-19	I went because in that time, I think that all the health posts, the hospitals – all of them – were practically closed because they only attended to people with COVID; they were closed.	Patient #2, male, 18-34 years old, pulmonary TB
	From the [public health system] I did not receive good care. I was waiting there almost half a day, and no one gave me any information. They did not want to help me because everything was totally COVID. So I went back to the [private health facility] where they were going to do surgery, and everything was expensive.	Patient #11, male, 18-34 years old, extrapulmonary TB
	I went directly to the [public hospital], waited in line and everything. Once the doctor in the general surgery area saw me, I asked her the situation – they had referred me from the clinic and told me to get a biopsy. She told me that because of COVID, everything was collapsed, and they were not doing this procedure.	Patient #16, male, 18-34 years old, extrapulmonary TB
	It is like in my case, I no longer knew what to do because going to a hospital, sometimes – the hospital right now is more looking out for people who are sick from the pandemic. So for me to go get examinations there is difficult.	Patient #21, female, 35-59 years old, pulmonary TB

Pulmonologists were not accessible because they were dedicated to treating COVID-19 patients	In my case as well, because there were not many specialists – they were all attending to COVID – so everyone went to general medicine. And well, general medicine is not the same as going to see a pulmonologist.	Patient #6, male, 18-34 years old, pulmonary TB
	In the hospital I was delayed so much because I could not get an appointment. Because one day they sent me for an appointment, but then there wasn't one. So I had to wait and re-book the appointment. Then they gave me a pulmonology appointment for [almost 2 months later], because there were no appointments, there were no doctors. Because of the pandemic, everything was restricted.	Patient #21, female, 35-59 years old, pulmonary TB
The private sector was more accessible, but the private sector only offered chest radiography, not a complete TB evaluation	I went because in that time, I think that all the [public] health posts, the hospitals, they were all were practically closed because they only attended to people with COVID; they were closed. I went to [a public-private partnership hospital], but it was closed – they would not see patients. And I went to one of those [private] medical centers around that area, and there they gave me a chest x-ray.	Patient #2, male, 18-34 years old, pulmonary TB
	**Interviewer**: Did they do a test on a sputum sample?<br>**Participant**: No, not a sputum test – still no.<br>**Interviewer**: But for COVID, to rule out COVID, did they do a molecular or serological finger-prick test?<br>**Participant**: It wasn't a finger-prick test – they told me it was a rapid test, nothing more. And it came back negative. But because of the chest x-ray, they said it was COVID.	Patient #6, male, 18-34 years old, pulmonary TB
	First, they sent me to get blood drawn, and then went to get a chest x-ray, and then they sent me to get a COVID test…The COVID test was negative. And the doctor looked at the chest x-ray and told me, "Your lungs look a certain way…there is something suspicious. It is possible that you have tuberculosis, so go to the nearest [public health center]."	Patient #8, female, 18-34 years old, pulmonary TB
	I went to a [private health center], where they asked me to get a chest x-ray. I did it, and the same day they looked at it; they looked at it and told me that these were just the effects of COVID.	Patient #18, female, 18-34 years old, pulmonary TB
Doctors did not want to give care because of fear that the patient had COVID-19	**Interviewer**: What do you think was the reason why as soon as you went there, they could not give you a diagnosis or at least some idea of what you had?<br>**Participant**: I think that mostly they would not because of fear, I assume of COVID. And because of this, they did not want…<br>**Interviewer**: They did not want to attend to you right there because…<br>**Participant**: Because this could be a symptom [of COVID].	Patient #14, male, 18-34 years old, extrapulmonary TB

Table S3: Example quotations showing how patients received COVID-19-related diagnoses, leading to delays and costs

Theme	Example quotation	Patient
TB and COVID-19 symptoms are similar	Because right now with fever – if it is fever, then it is COVID, right? At times we do not think about it, because there are different illnesses that can cause it.	Patient #5, female, 35-59 years old, pulmonary TB
	So this coronavirus disease, its symptoms are very similar to what I felt when I first got sick. For example, I had a high fever, I had difficulty breathing, and I had headaches and chills.	Patient #10, male, 18-34 years old, extrapulmonary TB
	[I had] a permanent cough, chest pain…I thought it was COVID, and because of this I went to the doctor quickly. But it was not COVID after all.	Patient #12, female, 35-59 years old, pulmonary TB
	I had fever all day, from when I woke up until I went to sleep. And the only thing I suspected was that I might have gotten COVID.	Patient #16, male, 18-34 years old, extrapulmonary
Doctors diagnosed or offered treatment for COVID-19 based on symptoms or abnormal chest radiography	I went to the doctor, and immediately the doctor said, "COVID," and gave me treatment for a week. But I continued with the cough, I continued with the pills, vials – nothing. And finally I returned to the doctor. The doctor said, "take a sputum test and get a chest x-ray."	Patient #12, female, 35-59 years old, pulmonary TB
	The doctor asked me, "what symptoms do you have?" And I said, "I have cough, fever, and it hurts a lot in my groin." And she said, "you don't have the symptoms necessary to do a test." She checked my lungs and said, "you are fine." And she said, "well, we are going to prescribe you treatment" – COVID treatment because it was ivermectin, paracetamol, ibuprofen.'	Patient #16, male, 18-34 years old, extrapulmonary TB
	I had a cough, so I self-medicated with pills, which got rid of my cough. My cough went away, and I was fine. After some months, I got worse. I got worse, the cough came back. This year was too much because I had gotten sick a lot, and I went to a [private health center], where they asked me to get a chest x-ray. I did it, and the same day they looked at it; they looked at it and told me that these were just the effects of COVID.	Patient #18, female, 18-34 years old, pulmonary TB
Patients let themselves be influenced by doctors even if they doubted that they had COVID-19	The only symptom I had until then was a cough that would not go away, it would not go away with anything. And that is why I went - I said, "Wow! I do not think this is COVID because I do not have a headache, body ache, or anything else." Well, I went, and the doctor prescribed me these medications for COVID – ivermectin and all these things. And well, I took them, but I went on in the same way – they didn't do anything for me. On the contrary, I think I got worse.	Patient #2, female, 18-34 years old, pulmonary TB
	I did not know that I had COVID. Because I did not have any of the symptoms. When they took my chest x-ray and they told me I had COVID, I was surprised because I did not have any of the symptoms.	Patient #18, female, 18-34 years old, pulmonary TB

Symptoms attributed to vaccine side effects	The doctor said that because they had made me get the vaccine when I had a cough, that this had damaged my bronchia.	Patient #21, female, 35-59 years old, pulmonary TB
	In the health centers they told me, "it could be that, it could be cysts, it could be a reaction to the vaccine" because I had gotten the two COVID vaccine shots.	Patient #26, female, 18-34 years old, extrapulmonary TB
Delay caused by treatment and isolation following a COVID-19 diagnosis	So they treated me [for COVID-19]. First it was a month of treatment, but every two weeks I had to do a chest x-ray to see the improvement, and every two weeks they changed the medications. That is how it went; I do not remember if it was two or three months. But finally, when I was just about to finish, because I was getting chest x-rays every two weeks, that is where the report stated that I had the beginnings of tuberculosis – that I had tuberculosis that was already advanced, or something like that.	Patient #6, male, 18-34 years old, pulmonary TB
	She told me, "We are going to consider that you have suspected COVID, and you will have to stay in your house. Do not go out for any reason. You will continue with the treatment, we will be calling you, monitoring you, to see how you are and what you are doing."	Patient #16, male, 18-34 years old, extrapulmonary TB
Patients paid for COVID-19 tests or treatments	Well, I took the treatment, but this treatment was extending to a week – that is, from three days to a week – and on top of that more medications, and on top of that, every time I had to pay	Patient #6, male, 18-34 years old, pulmonary TB
	They did a rapid test and my husband spent 70 soles [19 USD] there.	Patient #15, female, 18-34 years old, pulmonary TB

Table S4: Example quotations showing how patients felt that tactics for raising COVID-19 awareness should be applied to TB

Theme	Example quotation	Patient
Public awareness interventions should raise TB awareness and normalize TB screening, similar to what has been done for COVID-19	[Talking about TB screening] I would say "I've done my test," just like people get their COVID test now. COVID is well known. People go, and it is normal; people go and get their COVID test. This is how it should be [for TB], I think; it should be more talked about.	Patient #2, male, 18-34 years old, pulmonary TB
	They should make campaigns that are shown on television, just like there are for COVID. They should also do this for TB, which is a disease – they should explain what the symptoms are and everything, how it is treated.	Patient #8, female, 18-34 years old, pulmonary TB
	Everywhere there should be more specialists or an informational talk about what TB is. Because I think that people talk a lot about TB, now that there has been COVID. I think that it has increased, and there should be more talks so that people are more informed.	Patient #15, female, 18-34 years old, pulmonary TB
